# Resistance to fracture of intraradicular posts made of biological materials

**DOI:** 10.1186/s12903-020-01295-0

**Published:** 2020-11-03

**Authors:** Karine Taís Aguiar Tavano, Adriana Maria Botelho, Dhelfeson Willya Douglas-de-Oliveira, Antonio Ferreira Avila, Rudolf Huebner

**Affiliations:** 1 Department of Dentistry, Federal University of Jequitinhonha and Mucuri Valleys, Rua da Glória, 187, Centro, Diamantinna, MG 39100-000 Brazil; 2 Department of Mechanical Engineering, Federal University of Minas Gerais, Belo Horizonte, Brazil

**Keywords:** Intraradicular posts, Dentin posts, Biomaterials, Bovine posts

## Abstract

**Background:**

The aim was to analyze the fracture resistance of human teeth treated endodontically and restored with posts made of bovine dentin, human dentin, or glass fiber, and to evaluate the fracture pattern.

**Methods:**

Cylindrical posts of 1.5 mm in diameter cemented to the roots of human maxillary canines presented a length of 15 mm, cervical diameter of 5–5.5 mm in the mesiodistal direction, and 7–7.5 mm in the vestibule-palatal direction. The groups studied were: Group I—10 glass fiber posts; Group II—10 human dentin posts; Group III—10 bovine dentin posts (self-adhesive resin cement); and Group IV—10 bovine dentin posts (resin-modified glass-ionomer cements). The coronal part of tooth was restored with a standardized core build-up using composite. All of the groups were submitted to a compression force test and the resistance to fracture was verified using a universal testing machine. The fracture pattern was likewise evaluated.

**Results:**

The values of resistance to fracture were: 723.3N in group I, 561.5N in group II, 556.6N in group III, and, 613.27N in group IV. However, no statistically significant difference was observed among the groups. The fractures in groups I and II were most commonly found in the middle/apical third and were considered irreparable. For restored teeth in group III, half of the fractures appeared in the cervical third and were reparable. In group IV, all of the fractures were reparable, with the majority in the cervical thirds.

**Conclusion:**

Bovine dentin can be used as intraradicular post to substitute human dentin and glass fiber posts. The greater the malleability of the post, the greater the chances of survival of the teeth when subjected to fracture testing.

## Background

In healthy teeth, the distribution of occlusal forces occurs in a harmonious manner through the crown, the root canal, and the supporting periodontal tissues. Materials presenting different mechanical properties from those of the dental tissue can cause a high concentration of stress when they are placed in the root canal [1–3].

A weakening of the dental structure may be caused by structural modifications imposed by endodontic treatment, lateral force, removal of pulp vitality, and progressive diminishing of the hardness and strength of the tooth, as well as loss of coronal and radicular tooth structure [3, 4], which can, in turn, cause either a root or a coronal fracture.

The use of posts set in the inner portion of the root canal has been widely discussed in the literature, in which the only apparent point of consensus is the retention increasing of the coronal restoration [1, 2, 5]. The purpose of the posts is not related to reinforcing the structure, but rather to retaining and stabilizing the restorative materials. However, the hardness and resistance of the materials are parameters that influence the biomechanical behavior of the posts in the inner portion of the root canal [2, 6, 7].

The use of posts made of extracted human teeth (biological posts) represents a feasible option for intraradicular anchoring [8, 9]. They also offer the following advantages: low dentin stress preservation of the dentinal walls inside the root canal, biocompatibility, and adaptation to the root canal [8–10]. These characteristics allow greater resistance to the tooth and greater retention of the post, a resilience comparable to the natural tooth, and excellent adhesion to tooth and composite resin [8–10].

It has been found that human teeth are morphologically and histologically similar to teeth from other mammals [11]. The composition of human enamel and dentin is similar to the composition of bovine dental structures [12]. It has been reported that the adhesion to the superficial layer of dentin showed no significant difference between human and bovine dentin [11]. The morphology of coronal dentin and enamel is similar when comparing bovine and human teeth. Moreover, bovine teeth provide other advantages, such as greater availability, larger dimensions, and radiodensity similar to human dentin [11–13]. In addition, ethics committees around the world have stimulated the replacement of human teeth with animal ones for researches purposes [11]. Currently, there is a constant search for an ideal material to anchor coronal restorations [14, 15].

Therefore, the present study aims to analyze the fracture resistance of human teeth treated endodontically and restored with posts made of bovine dentin, human dentin, or glass fiber dentin, and then evaluate the fracture pattern and whether or not it is reparable.

## Material and methods

### Prefabricated glass fiber posts

Ten cylindrical prefabricated glass fiber posts were acquired from Reforpost® (Angelus, Paraná, Brazil), with 1.5 mm in diameter and 15 mm in length, which were used as a control group.

### Manufacture of biological posts

This study was approved by the Human Research Ethics Committee under protocol number 065/09 and by the Animal Experimentation Committee of the Federal University of Minas Gerais. Twenty bovine teeth from slaughterhouses and 50 human extracted teeth, duly donated, were acquired. These teeth were sterilized for 7 days in a 10% formaldehyde solution [16] and maintained in distilled water during all stages of the study.

**Fig. 1:**
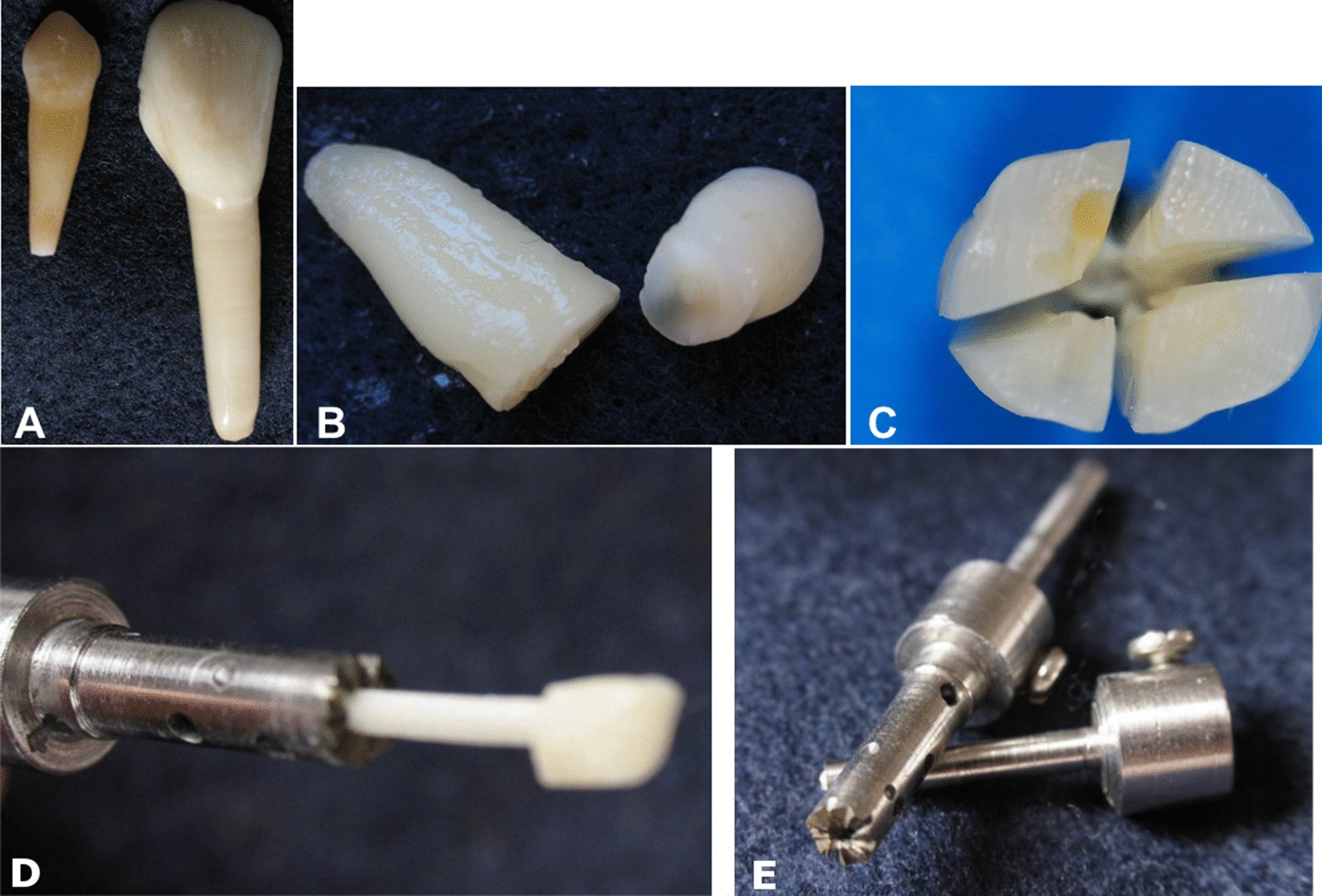
**a** Human canine and bovine incisor; **b** root and crown separated by bur; **c** root divided into four parts; **d**, **e** drill

The biological posts were created from the roots of bovine incisors and human canines (Fig. 1) using a drill specially designed and made for this study (Fig. 1), which standardized the specimens in a cylindrical form.

All of the teeth had their coronal portion cut by a cylindrically-shaped diamond tip burr at high rotation under cooling, with this tooth portion being subsequently discarded (Fig. 1). The root canal portion was sectioned along the long axis, in four parts (Fig. 1), with a carborundum disc at low rotation under cooling.

#### Bovine posts

**Fig. 2:**
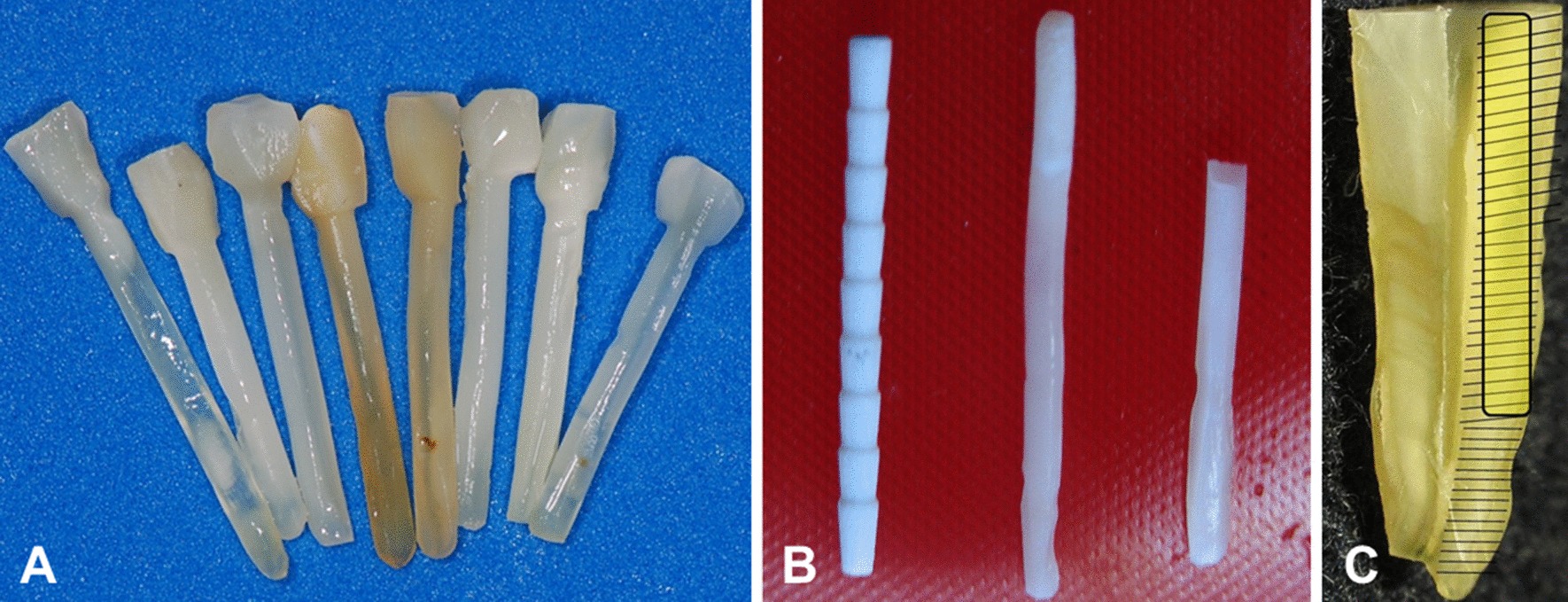
**a** Biological posts; **b** glass fiber, human dentin and bovine dentin posts; **c** root total length and post drawing according to scale

Although each bovine root provided four posts, only one post was made from each root. Twenty cylindrical posts of 13 mm length were configured with a cylindrical intraradicular portion of 10 mm length (1.5 mm in diameter), and a cylindrical coronal portion of 3 mm length (2 mm in diameter) (Fig. 2).

#### Human posts

Ten human maxillary canines were cut out, acquiring one post per root, totaling 10 biological posts made of human root dentin. They presented the same proportions as the bovine posts (Fig. 2).

The dimensions of both bovine and human posts were in accordance with the size of the prefabricated glass fiber post in order to standardize the posts.

All procedures performed in studies involving extracted human teeth were in accordance with the ethical standards of the institutional and/or national research committee (Research Ethics Committee of Federal University of Minas Gerais, protocol 065/09) and with the 1964 Helsinki declaration and its later amendments or comparable ethical standards. The subjects signed an informed consent form prior to the study beginning.

### Specimen preparation

Forty human canines were selected and placed in four experimental groups (n = 10/group) for the fracture test. The teeth had their crowns removed, and endodontic treatment was performed. Briefly, the root canals of all groups were prepared chemomechanically. The working length of the roots was determined by the visual method by introducing a file #15 (Maillefer, Dentsply, Rio de Janeiro, Brazil), inside the root canal until it reaches the foramen, then backing up one millimeter. It was adopted the modified classical technique until the file #80 (Maillefer, Dentsply, Rio de Janeiro, Brazil). After intermittent rinsing with 2.5% sodium hypochloride, the roots were dried with paper points (Maillefer, Dentsply, Rio de Janeiro, Brazil), and the roots were obturated with laterally condensed gutta-percha (Maillefer, Dentsply, Rio de Janeiro, Brazil) and an endodontic cement (Sealer 26®, Dentsply, Rio de Janeiro, Brazil).

The teeth presented a mean root length of 15 mm, with a cervical diameter of 5–5.5 mm in the mesiodistal direction and 7–7.5 mm in the vestibular-palatal direction. These were sterilized by immersion in a formaldehyde solution for 7 days and maintained in distilled water until the beginning of the experimental procedures.

To simulate the periodontal ligament, each tooth was marked with a pen at a distance of 2.0 mm below the cementoenamel limit, covering 13 mm of the root. This area was covered by a #7 wax (Wilson, São Paulo, Brazil), liquefied in a water bath until reaching its demarcation line, at approximately 0.3 mm in thickness.

**Fig. 3:**
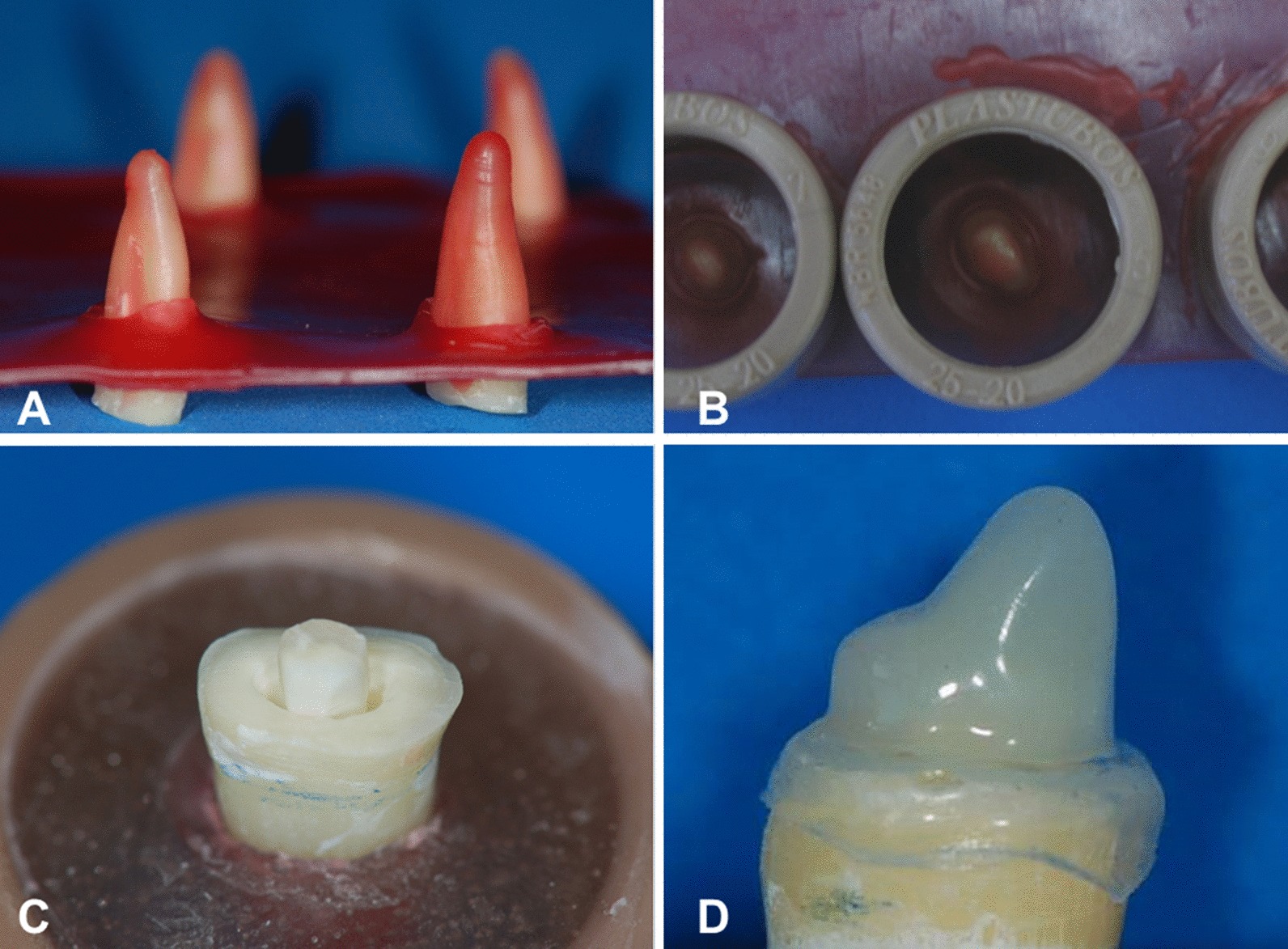
**a** Roots stuck to the wax slide; **b** PVC tubes stuck to the wax slide; **c** root with biological post; **d** human canine prepared for the total crown and polypropylene matrix

On a #7 wax plate (Wilson, São Paulo, Brazil), eight punctures were made at equal distances from each other. In each puncture, a root was set in the area marked by the liquefied wax (Fig. 3). Polyvinyl chloride (PVC) cylinders (Tigre, São Paulo, Brazil) of 25 mm in diameter were also stuck to the wax and placed over the exposed roots, with one of its borders having been previously heated (Fig. 3).

Next, the chemically activated acrylic resin was applied in the inner portion of the PVC cylinder and the elements were maintained in distilled water. Two hours later, they were removed from the wax plate. The teeth were removed from the artificial alveoli in acrylic resin and cleaned for posterior simulation of the periodontal ligament. The preparation of the roots and the insertion of the PVC tube was performed 24 h after the biological posts were made, time in which the posts were conserved in distilled water.

To standardize the coronal portion of the post (nucleus), a preparation of the total crown was performed on a healthy canine with the same dimensions of the teeth from the present study. The coronal portion presented 5 mm height and 1 mm beyond the shoulder line (Fig. 3). This procedure was then duplicated using additional silicone (EXPRESS, 3 M ESPE, USA), and a special stone plaster was applied to the molds to create the plaster models of the prepared nucleus. After the crystallization of the plaster (Vigodent, Coltene, Brazil), the polypropylene matrixes were created in a vacuum plastifying machine (FGM, Brazil).

### Post cementation

Four experimental groups (n = 10) were created for testing:

- *Group I:* control group, Reforpost® glass fiber cylindrical; self-adhesive cement resin with dual polymerization, Rely X U-100 (3M ESPE, USA).
- *Group II:* biological cylindrical posts made of human dentin; self-adhesive cement resin with dual polymerization, Rely X U-100 (3M ESPE, USA) (3M ESPE, US).
- *GROUP III:* biological posts made of bovine dentin; self-adhesive cement resin with dual polymerization, Rely X U-100 (3M ESPE, USA).
- *GROUP IV:* biological posts made of bovine dentin; resin-modified glass-ionomer cements, RelyX™ Luting 2 (3M Espe, USA).

All of the root canals had their fillings partially removed (10 mm), were widened to the size of a Largo 6 drill (1.6 mm in diameter), and then cleaned with 24% EDTA (Biodinâmica Ltda., Paraná, Brazil) for 3 min, followed by abundant rinsing with distilled water for 1 min and dried with absorbent paper cones (Dentsply, USA).

For the cementation stage, each glass fiber post was cleaned with 70% alcohol and dried. The biological posts were rinsed with an air/water spray and dried with absorbent paper. Each post received a fine layer of cement along its border and was immediately placed in the root canal slowly. After excess removal, the cement was photopolymerized for 40 s, followed by a waiting period for the final chemical polymerization to be achieved.

The portion of the post that remained outside the root was maintained, together with the composite resin, to characterize the nucleus. Using the polypropylene matrixes obtained in a vacuum plastifying machine, the form and dimension of the coronal portion of the nuclei (5 mm in height and shoulder of 1 mm) were standardized for all groups (Fig. 3).

To accomplish this, after complete polymerization of the cements, the adhesive procedures were performed equally for all groups to formulate the nucleus, together with the matrix and the composite resin. Application of 37% phosphoric acid for 15 s on the post and dentin, abundant rinsing, and drying with absorbent paper; application of the Single Bond adhesive system (ESPE–USA) on the entire dentin and post, and polymerization for 20 s with a light-emitting diode (wavelength of 470 nm); placing of the Filtek™ Z250 composite resin (3 M ESPE, Sumaré, Brazil) around the cemented post; polymerization for 40 s on all sides; followed by removal of the excess material using diamond tip burrs.

Each root and artificial alveoli made of acrylic resin was cleaned, all wax removed and dried with absorbent paper. The specific adhesive of the simulator material (Polyether Adhesive, 3M ESPE, Germany) was attached to the roots and inside the artificial alveoli, waiting 15 min for it to completely dry. Next, the impression material made of polyethylene from Impregum (3M ESPE, Germany) was handled, according to manufacturer instructions, and placed in the artificial alveoli by means of a polyethylene syringe [17]. The tooth was then re-implanted upon this, removing the excesses [17].

The teeth were stored for 24 h in 100% humidity [17] to perform the 135° compression tests.

### In vitro fracture resistance test

**Fig. 4:**
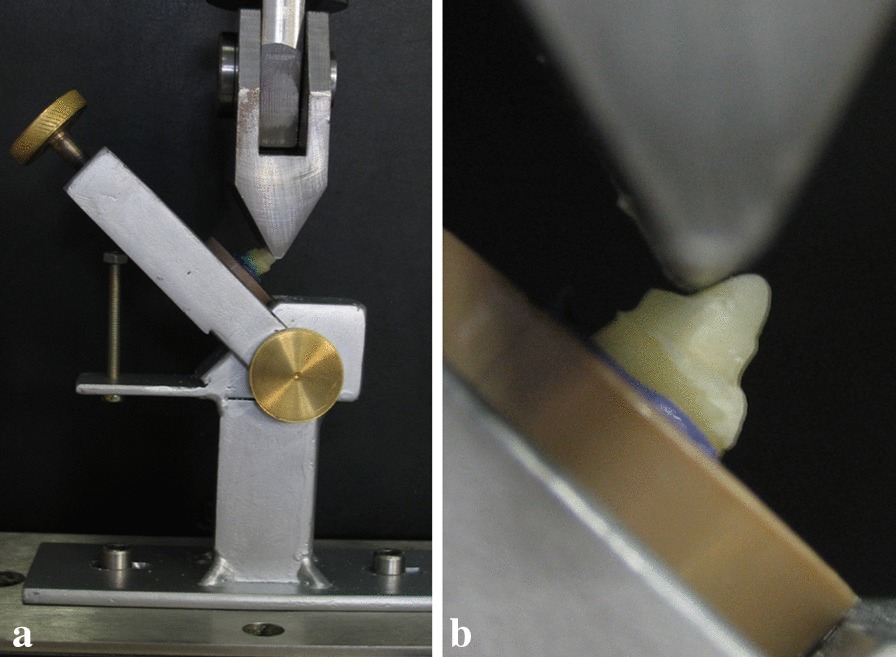
**a** Device for 135° fracture test; **b** detail of the active point at 135° angle with the long axis of the tooth

Compressive loads were applied in an EMIC universal testing machine (Instron Brasil Equipamentos Científicos Ltda, Paraná, Brazil) at a velocity of 0.5 mm/min, in the palatal region of the specimens, at a 135° angle in relation to the long axis of the tooth (Fig. 4), until fracture.

**Fig. 5:**
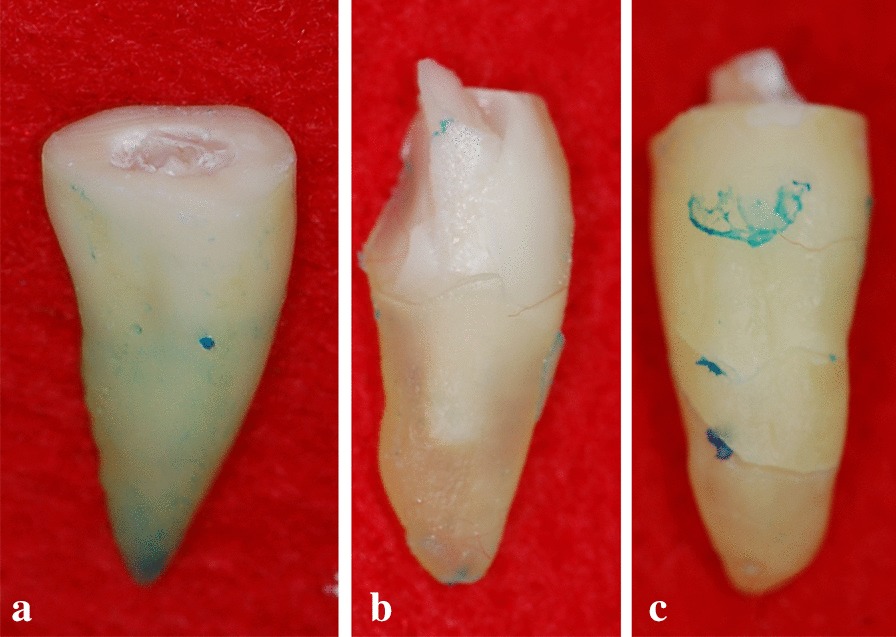
Types of fracture. **a** Cervical fracture; **b** middle third fracture; **c** apical fracture

These fractures were classified according to the tooth region (cervical, middle, or apical) (Fig. 5) and whether or not they were reparable [18]. Dental feasibility was considered to characterize the repair, either by clinical crown lengthening or by apicectomy, aimed at the maintenance of appropriate bone support [18].

### Statistical analysis

The data were analyzed by the Minitab® 15 statistics software. Descriptive analyses were performed to provide mean, standard deviation, and frequencies. The data were then submitted to the normality test. The differences in the fracture resistance values were verified by means of Analysis of Variance (One-Way ANOVA). The distribution of the fracture patterns among the groups were verified by Chi-Square test. A significance level of 5% was adopted.

## Results

In group I, two teeth presented fractures classified as reparable, located in the cervical third. It was observed that roots fractured in many places and that the composite resin cement had not properly adhered to the root wall. In group II, four teeth presented fractures classified as reparable, located in the cervical third. In group III, the fractures were classified as reparable in five teeth (cervical third). In group IV, the fractures were classified as reparable in all teeth. A statistically significant association in the fracture distribution was observed (Table 1).

**Table 1:** Distribution of the fracture pattern by group

	Group I n (%)	Group II n (%)	Group III n (%)	Group IV n (%)	p*
Cervical third	2 (20.0)	4 (40.0)	5 (50.0)	9 (90.0)	
Middle third	0 (0.0)	0 (0.0)	0 (0.0)	1 (10.0)	0.017
Apical third	8 (80.0)	6 (60.0)	5 (50.0)	0 (0.0)	

*Chi-square test

No statistically significant difference was observed in the fracture resistance when the type of posts was compared (Table 2).

**Table 2:** Values of fracture resistance by group

Group	Mean (N)	SD	p*
Group I	723.30	253.06	0.298
Group II	561.55	194.13	
Group III	556.60	194.99	
Group IV	613.27	220.62	

*SD* standard deviation

*ANOVA one-way

## Discussion

The reconstructive dental materials must contain properties that ensure the integrity of the teeth when confronting masticatory or parafunctional forces, in turn allowing them to be subtly dissipated through dental substrates [13, 19]. The present study attempted to evaluate the fracture resistance of biological and glass fiber posts cemented to human teeth. The glass fiber posts and biological posts were shown to be useable as an anchor in endodontically treated teeth.

As every scientific investigation must approximate the in vitro assay to the in vivo reality, it was necessary to simulate the periodontal ligament. Considering that the viscous behavior of the periodontal ligament is responsible for the dissipation of loads [20], this simulation was performed using high-density polyethylene with the appropriate consistency to execute this function [17]. This allowed the simulation of tooth movement within the alveolus and, consequently, the dissipation of the force from the mechanical assay. This is also of paramount importance, considering the growing number of computer-simulated studies that apply the method of finite elements [21].

In the same way, the 135° angle was chosen to apply the force, as it simulates the maxillary/mandibular occlusal relationship of Angle Class I individuals in the anterior region [22]. It is important to note that the more precise the simulation of the clinical situation, the better generalized result to dental practice, where all structures must be analyzed [5, 6].

Irreparable fractures consistently occurred when the composite resin cement was present. The RelyX U100 self-adhesive resin cement contains bifunctional groups of methacrylate (phosphorylated methacrylate), which, due to its acidic mature, allows demineralization and posterior infiltration within the dental surface, resulting in micromechanical retention [23, 24]. As such, it can be suggested that an improper adhesion of the cement to the root dentin led to adhesive failures and irreparable fractures in the middle/apical third. Moreover, the greater modulus of elasticity (70.86 MPa) of this cement [24] also played a key role, causing small elastic deformations and, consequently, fractures.

The GIC itself is a widely used material in dentistry today, mainly due to its characteristics of biocompatibility, dental adhesion, and release of fluoride [25]. When the Rely X Luting GIC was employed, fractures occurred in the cervical and middle thirds. This finding is in accordance with other studies that have analyzed the modulus of elasticity of this material, which is considered to most resemble human dentin [26, 27]. The resin-modified glass-ionomer cements presented the greatest flexural resistance and modulus of elasticity when compared to the self-adhesive resin cements [26–28]. The mechanical properties of the GIC may explain the reparable characteristics of the fractures.

Comparing the values found herein with the maximum biting force of anterior teeth (men 194 N and women 153 N) [29–31], it can be stated that the biological posts were able to withstand physiological masticatory forces. It is important to note that the biting force of patients who grind their teeth can be up to 6 times greater than in those who do not [32].

Although the statistical analysis showed no difference among the biological posts used regarding fracture resistance strength, the worst fracture conditions were observed in groups I and II, both using self-adhesive resin cement. It can be noted that the composite resin cement in the fracture area had not adhered to the root dentin, but rather to the posts, which may explain the high prevalence of irreparable fractures. Thus, the hardness of the posts combined with the hardness of the composite resin cement, forming a unique, more resistant block than the remaining root. Achieving this adhesion at deeper level in the root canal is a recurring difficulty and a challenge in dentistry today. Yang et al. [23] observed that the degree of polymerization of the composite resin cement is an important factor in adhesive resistance.

Comparing the fracture types between both groups of bovine dentin posts, the difference lies in the kind of fracture. When glass ionomer cement is used, the dissipation of the forces seemed to be more harmonious, causing fractures near the cervical area where the force was applied as compared to those cases cemented with composite resin.

Biological posts, which have the same resilience as the remaining tooth, ensure the advantage of not causing stress to the dentin [8, 9]. Their main objective would be a restoration in “monoblock” form [8], that is, through adhesion, to form a unique biomechanical compound between the dental structure and the restorative materials (biological post, cementing agent, and root canal dentin). This fact may explain the good fracture resistance presented by both bovine and dentin posts.

The association of the bovine dentin posts, which contains an elasticity modulus (8.91 GPa) close to that of human dentin (9.51 GPa) [33, 34], with a cement that has flexural elasticity modulus (31.6 MPa, manufacturer data) lower than those of root dentin, results in fractures that are more favorable to repair. Furthermore, the cement has chemical adhesion to both posts and root canal dentinal substrate. These factors together may ensure the integrity of teeth weakened by endodontic treatment.

The association between bovine dentin posts and resin modified glass ionomer cement also presented reparable fractures. Clinically, this is what is expected of an intraradicular anchoring, i.e., the posts should be able to resist the masticatory force and, if failed, this failure should be reversible in order to preserve the tooth.

Actually, glass fiber posts present the best properties when compared to the other non-biological posts, justifying their use on a broad scale [35]. They are most closely resemble the dentin as regards the modulus of elasticity and are, therefore, bioacceptable, as well as causing reparable fractures when submitted to extreme forces.[1, 35, 36]. In contrast, Plotino et al. [37] and Toksavul et al. [38] observed that glass fiber posts caused fractures that were considered irreparable, which is in accordance with the present study where the worst and irreparable fracture patterns were observed when using this type of post.

No other restorative material is better than the mechanical and aesthetic properties of the dental structure [8, 9, 34], which can be found in the biological posts. However, the disadvantages in using them include the lack of human tooth banks and slight difficulty in their preparation. In addition, the patient receiving a biological post must be told about the origin of the posts, the sterilization process, biosafety standards, and sign an authorization form as well. It is also important to tell to the patient that the biological posts will not be exposed to the oral environment. Most patients accept this treatment without restrictions when so informed [8, 9]. Some patients, however, reject this treatment due to the fact that the tooth had been extracted from an unknown person. Another common limitation is the further need for endodontic re-treatment. Considering that the tooth is similar to a tooth containing an obliterated root canal [9], the removal of this biological post will be performed by re-opening the root canal with dental drills.

The present resistance to fracture in 135° compression tests is similar to those reported in the literature with similar methodology, but with other types of posts and cementing materials [8, 15, 36]. Kaizer et al. [8] analyzed biological posts made from human teeth roots cemented with composite resin cement, in different impact situations, and concluded that the teeth with conventional root canal preparation presented better resistance to fracture, with values of 533 N. They also concluded that biological posts are capable of promoting a reinforcement of the roots when these are not completely destroyed.

It is important to note that the present specimens were tested mechanically without crowns. Considering the studies that have used a similar methodology, the values of the fracture results reported herein were higher than those related by Giovani et al. [36] (16.75–27.4 N) and by Frater et al. [39] (154.5–240.6 N). The present fracture values are in agreement with those of Fadag et al. [40], who reported resistance fracture loads ranging from 524 (N) to 764 (N). M oreover, a study with a dentin post showed high fracture resistance (793.1 N) [41], even higher than those reported herein.

The use of a bovine post as an intracoronary retention aid should be encouraged by clinicians since the post used in this study presented satisfactory fracture resistance, was easy to obtain, biosafe (after sterilization), and both histochemically and anatomically similar to human teeth [11, 12].

Thermomechanical aging did not affect the fracture resistance of the post or the core build-up systems [42]. For this reason, and considering that the influence of the post systems on fracture resistance of roots was the primary aim of the present investigation, no thermo-mechanical cycling was used herein.

It is important to note that the cement was applied to the post surface alone to better simulate clinical practice. A limitation of the present study was that cement was not applied to the root canal with small tips.

Nonetheless, new studies are warranted to confirm the results obtained herein, and determine the most reliable methods of sterilization that will not intervene in the mechanical properties of this substrate. This would allow the materials to be used clinically, without restrictions, and would solve the issue of seeking the ideal material for intraradicular retention. Moreover, the measure would not only favor the retention itself, but would also reinforce the weakened dental structures.

## Conclusion

It can be concluded that prefabricated glass fiber posts, bovine dentin, and human dentin posts presented similar values of fracture resistance in cases of endodontically treated human teeth. The present findings suggested that both bovine and human dentin posts can be used as an intraradicular post. The bovine posts demonstrated satisfactory results when cemented with glass ionomer cement, being an alternative biological post, given the difficulty in acquiring human teeth. About half of the fractures occurred in the cervical area in a reparable form.

## Data Availability

Data will be made available upon request by email.

## Abbreviation

PVC: Polyvinyl chloride
